# Effects of forest cover on richness of threatened fish species in Japan

**DOI:** 10.1111/cobi.13849

**Published:** 2021-12-02

**Authors:** Edouard Lavergne, Manabu Kume, Hyojin Ahn, Yumi Henmi, Yuki Terashima, Feng Ye, Satoshi Kameyama, Yoshiaki Kai, Kohmei Kadowaki, Shiho Kobayashi, Yoh Yamashita, Akihide Kasai

**Affiliations:** ^1^ Field Science Education and Research Center (FSERC) Kyoto University Kyoto Japan; ^2^ Research and Educational Unit for Studies on Connectivity of Hills, Humans and Oceans Kyoto University Kyoto Japan; ^3^ Laboratoire des Sciences de l'Environnement Marin (LEMAR), Institut Universitaire Européen de la Mer (IUEM) Université de Bretagne Occidentale Plouzané France; ^4^ Plastic@Sea Banyuls‐sur‐Mer France; ^5^ Faculty of Fisheries Sciences Hokkaido University Hakodate Japan; ^6^ Biodiversity Division National Institute for Environmental Studies (NIES) Tsukuba Japan; ^7^ The Hakubi Center for Advanced Research Graduate School of Agriculture, Kyoto University Kyoto Japan

**Keywords:** anthropogenic activity, coastal water bodies, environmental DNA metabarcoding, estuary, land use, actividad antropogénica, cuerpos de agua costeros, estuario, metasecuenciación de ADN ambiental, uso de suelo

## Abstract

Estuaries––one of the most vulnerable ecosystems globally––face anthropogenic threats, including biodiversity loss and the collapse of sustainable fisheries. Determining the factors contributing to the maintenance of estuarine biodiversity, especially that of fish, is vital for promoting estuarine conservation and sustainability. We used environmental DNA metabarcoding analysis to determine fish species composition in 22 estuaries around Japan and measured watershed‐scale land‐use factors (e.g., population size, urban area percentage, and forest area percentage). We sought to test the hypothesis that the richness of the most vulnerable estuarine fish species (i.e., registered by the Japanese Ministry of the Environment in the national species red‐list) is determined by watershed‐scale land‐use factors. The richness of such species was greater, where forest cover was highest; thus, forest cover contributes to their conservation. The proportion of agriculture cover was associated with low species richness of red‐listed fishes (redundancy analysis, adjusted *R* ^2^ = 43.9% of total variance, df = 5, *F* = 5.3843, *p* = 0.0001). The number of red‐listed species increased from 3 to 11 along a watershed land‐use gradient ranging from a high proportion of agriculture cover to a large proportion of forest cover. Furthermore, the results showed that throughout Japan all the examined watersheds that were covered by >74.8% forest had more than the average (6.7 species per site) richness of red‐listed fish species. This result can be attributed to the already high average forest cover in Japan of 67.2%. Our results demonstrate how the land use of watersheds can affect the coastal sea environment and its biodiversity and suggest that proper forest management in conjunction with land‐use management may be of prime importance for threatened fish species and coastal ecosystems in general.

## Abstract 2

*Article impact statement*: Agricultural and forest cover have opposite effects on the richness of red‐listed fish species in Japanese estuaries.

## Abstract (es)

Efectos de la Cobertura Forestal sobre la Riqueza de Especies Amenazadas de Peces en Japón

**Resumen:**

Los estuarios—uno de los ecosistemas más vulnerables a nivel mundial—enfrentan amenazas causadas por el hombre, incluyendo la pérdida de biodiversidad y el colapso de las pesquerías sustentables. La determinación de los factores que contribuyen al mantenimiento de la biodiversidad estuarina, especialmente la de los peces, es vital para fomentar la conservación y sustentabilidad estuarinas. Usamos un análisis de metasecuenciación de ADN ambiental para determinar la composición de especies de peces en 22 estuarios de Japón y medimos los factores del uso de suelo a nivel de cuenca (p. ej.: tamaño poblacional, porcentaje de área urbana y porcentaje de área forestal). Buscamos probar la hipótesis de que la riqueza de las especies de peces más vulnerables (es decir, aquellas registradas por el Ministerio Japonés del Ambiente en la lista roja de especies a nivel nacional) está determinada por los factores de uso de suelo a nivel de cuenca. La riqueza de dichas especies fue mayor en donde la cobertura forestal era la más alta; por lo tanto, la cobertura forestal contribuye a la conservación de estas especies. La proporción de la cobertura agrícola estuvo asociada con una baja riqueza de especies de peces en la lista roja (análisis de redundancia, *R* ^2^ ajustada = 43.9% de la varianza total, gl = 5, *F* = 5.3843, *p* = 0.0001). El número de especies en la lista roja incrementó de 3 a 11 a lo largo del gradiente de uso de suelo de la cuenca, yendo desde una proporción alta de cobertura agrícola a una proporción alta de cobertura forestal. Además, los resultados mostraron que, en Japón, todas las cuencas analizadas que contaban con una cobertura forestal >74.8% tenían mayor riqueza de especies de peces en la lista roja que el promedio (6.7 especies por sitio). Este resultado puede atribuirse a la ya de por sí elevada cobertura forestal de Japón (67.2%). Nuestros resultados demuestran cómo el uso de las cuencas puede afectar al ambiente costero marino y a su biodiversidad y sugiere que la gestión adecuada de los bosques en conjunto con el manejo del uso de suelo puede ser de suma importancia para las especies amenazadas de peces y los ecosistemas costeros en general.

## INTRODUCTION

Coastal areas and estuaries are social‐ecological systems that provide vital services to humans and must be protected from increasing human demands on natural resources and from global changes in climate, elemental cycling, and economic systems (Tett et al., 2011). Furthermore, such areas have been places of human settlement for millennia (Marincioni & Negri, 2020). In 2017, nearly 2.4 billion people, representing 31% of the global population, lived within 100 km of a coastline (United Nations, 2017). As a result, coastal ecosystems, especially estuaries, are facing serious anthropogenic threats, including habitat modification or loss, invasive species, eutrophication, pollution, and overexploitation, that compromise their ecological integrity (Kennish, 2002; Simpson & Sharples, 2012; Yoshimura et al., 2005). Human activity and climate change have rapidly transformed ecosystems worldwide (Cloern et al., 2016), leading to a sharp decline in biodiversity and an increase in the number of species at risk of extinction, for example, red‐listed species (Rodríguez et al., 2015). Determining the factors contributing to the persistence of fish biodiversity is crucial for its conservation in estuaries and for understanding how best to develop and maintain sustainable coastal fisheries.

One of the key challenges associated with identifying factors determining estuarine biodiversity is that estuarine ecosystems are affected not only by environmental changes in the coastal zone itself, but also by environmental changes occurring in the entire watershed located upstream of the coast (Deiner et al., 2016). These propagating effects arise because various materials are transported through rivers downstream of the entire catchment basin (Dauer et al., 2000). These materials provide matter and energy to the aquatic trophic food web through detritus (e.g., leaves, fruits, and insects [Correa & Winemiller, 2018; Odum & Heald, 1975]), nutrients (e.g., nitrogen, phosphate, and silicate [Shimizu et al., 2014]), sediments, and contaminants (Allan, 2004; Shimizu et al., 2014; Sponseller et al., 2001). Furthermore, land‐use effects can be cumulative and interactive in exerting an influence on coastal ecosystems. For example, temperature changes caused by climate change (Lavergne et al., 2015), the introduction of invasive species (Gallardo et al., 2017; Walther et al., 2009), and dams (Cote et al., 2009) may cumulatively lead to erosion and affect aquatic stream diversity and ecosystem services. Thus, evaluating the effects that propagate through river flow from upstream of the watershed requires the integration of large‐scale, socioecological ecosystem data, ranging from estuarine, human‐populated, and urbanized lowland areas to mountains.

Recent studies show that environmental DNA (eDNA) metabarcoding is a highly useful tool for assessing biodiversity in aquatic ecosystems (e.g., Miya et al., 2020; Shaw et al., 2016; Thomsen et al., 2012a). This genetic approach is noninvasive, leads to minimal habitat disruption, and enables a faster and more effective yet complementary species identification than conventional net samplings or visual observations (e.g., Deiner et al., 2016; Nakagawa et al., 2018; Stoeckle et al., 2017), which are often too laborious or unfeasible to compare diversity across sites due to the differences in sampling methods and efforts (Pasquaud et al., 2015; Vasconcelos et al., 2015). Owing to the development of eDNA metabarcoding, the number of eDNA studies assessing estuarine biodiversity has rapidly increased worldwide (e.g., Goutte et al., 2020; Schwentner et al., 2021; Stoeckle et al., 2017). Although the eDNA approach is particularly advantageous for detecting inconspicuous or low‐abundance threatened species (Sigsgaard et al., 2015; Thomsen et al., 2012b; Wilcox et al., 2013), most eDNA metabarcoding studies on the diversity of fish species have not focused on threatened species and have generally been restricted to a few stream locations (Ahn et al., 2020; Doi et al., 2021). Kume et al. (2021) first reported a large‐scale pattern of estuarine and coastal fish communities around the Japanese archipelago based on eDNA metabarcoding at 25 estuaries and surrounding coasts. They reported that latitude and water temperature affected both river mouth and coastal fish communities but did not focus on the species at risk.

Japan is a mountainous country with high forest cover (67.2% [Japan Forest Agency, 2017]) and a high population density (340.8/km^2^ in 2015 [Ministry of Internal Affairs & Communication, Statistics Bureau of Japan, 2020]). Japan has a narrow topography from north to south, with mountainous areas at its center. Thus, short rivers flow in comparatively small watershed areas. Therefore, Japan is a suitable area for studying the effects of anthropogenic activity on fish species diversity in a watershed unit.

We sought to test the hypothesis that richness of estuarine red‐listed fishes, which are supposedly vulnerable to anthropogenic environmental disturbance, is determined by watershed‐scale land‐use factors. For this purpose, we compiled fish eDNA metabarcoding data and socioecological and physicochemical data in 22 watersheds (i.e., sites) in Japan and examined whether environmental parameters and land‐use affect the ability of estuaries to harbor red‐listed species.

## METHODS

### Study sites, water sampling, and eDNA analyses

Japan has 109 first‐class rivers (Ministry of Land, Infrastructure, Transport & Tourism, 2019). Among these, we selected the longest river (<150 km) in each of the 47 prefectures to eliminate extremely large rivers and unify the scale of the targeted watersheds. Thus, we focused on the estuarine portion of rivers with an average salinity between high and low tides of <20 at the time of sampling (*n* = 22). This ensured that fish species composition did not consist exclusively of marine species, of which there are fewer threatened species relative to freshwater fishes (IUCN, 2020).

**FIGURE 1:**
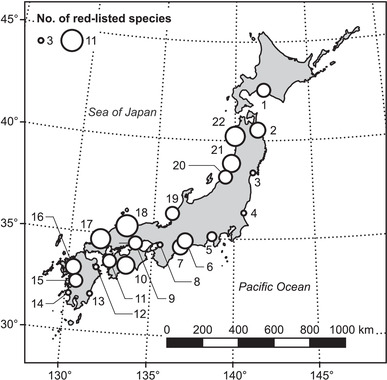
Map of water sampling locations in Japan. The size of circle is proportional to the number of fish species on the Japanese Red Lists detected at each site. Numbers are rivers: 1, Mukawa; 2, Mabechi; 3, Naruse; 4, Naka; 5, Fuji; 6, Yahagi; 7, Miyagawa; 8, Yamato; 9, Asahi; 10, Niyodo; 11, Hijikawa; 12, Onogawa; 13, Oyodo; 14, Sendai; 15, Kuma; 16, Chikugo; 17, Takatsu; 18, Hino; 19, Kuzuryu; 20, Arakawa; 21, Akagawa; 22, Yoneshiro

Surface water samples were collected from 22 major estuaries across Japan from June to August 2018 (Figure 1). Sampling was performed at both high and low tides to detect the maximum number of fish species (Kelly et al., 2018). The bucket used to collect samples was cleaned three times with Milli‐Q water and detergent between each sampling site to prevent contamination between samples. Whenever possible, we filtered up to 1 L of water from at least three different surface bucketthrows at each river site and tide with a 0.45‐µm polyethersulfone membrane Sterivex filter unit (Merck Millipore, Burlington, MA, USA) attached to a 50‐mL syringe. New filters and syringes were used for each sample. After filtration, the filter was immediately filled with 1.6 mL of RNAlater (Thermo Fisher Scientific, Waltham, MA, USA) to ensure complete membrane coverage. At each site, 500 mL of pure water was filtered and used as a negative control. All filter units were kept on ice during transport to the laboratory and stored at −30 °C until eDNA extraction.

Total DNA was extracted from the Sterivex filter units with a DNeasy Blood and Tissue Kit (Qiagen, Hilden, Germany), following the procedure described by Miya et al. (2016) and the manufacturer's protocol with minor modifications. Samples were sent to the Kazusa DNA Research Institute (Chiba, Japan) for paired‐end library preparation and next‐generation sequencing (MiSeq) as detailed by Miya et al. (2015). Data preprocessing and analysis of MiSeq raw reads were taken using a pipeline (MiFish 2.3) specially developed from four runs in USEARCH 10.0.240 (Edger, 2010). The minimum threshold for inclusion of a species in a sample was 16 reads. Few fish species were detected in the negative control samples, albeit within a small number of reads. The number of reads corresponding to every fish species detected in the negative control was deducted from the number of reads of the respective fish species detected in each sample. Detailed procedures for the extraction and verification of the species are described in the report by Ahn et al. (2020).

Census, land‐use, and physicochemical data sets of Japanese watersheds and rivers are publicly available online but are mostly in Japanese. The source code of the main tools, scripts, and algorithms used is available from https://github.com/edouard‐lavergne/eDNA‐Japan.

### Red Lists

The Japanese Ministry of the Environment (JMOE) compiled a red list of marine fish species (Ministry of the Environment, 2017) and a red list of brackish and freshwater species (Ministry of the Environment, 2019). The lists’ threat categories and definitions of those categories matched those of the International Union for Conservation of Nature (IUCN) Red List (IUCN, 2020): near threatened (NT), vulnerable (VU), endangered (EN), and critically endangered (CR). The exception is that the Japanese lists include the category endangered local population (LP). Species listed as LP have populations regionally isolated with a high risk of extirpation (Ministry of the Environment, 2017, 2019). Some species are not listed in the same categories on the IUCN and Japanese lists because of differences in threat levels at global and national scales. Because we focused on Japan, we used the JMOE lists.

Species that were detected using eDNA metabarcoding that were not included in the JMOE list were designated as least concerned (LC) so as to include all available species in the analyses. We refer to species on the JMOE list (i.e., non‐LC species) as *red‐listed species* and to species belonging to LP, VU, EN, or CR categories as *threatened species*. We then combined presence‐absence data obtained from eDNA metabarcoding with the JMOE lists (Appendix S1) to generate a matrix indicating the number of species per category in each estuary. We used *spp*. with a genus name to mean more than one species was detected and all species in the genus were in the same threat category.

### Watershed variables

The total watershed area, human population (number of inhabitants), and density data from 2015 were compiled for each watershed based on publicly available data (Appendix S2). Land‐use data from 2014 (i.e., geographic area of rice cultivation, agriculture not used for rice cultivation, forest, abandoned land, urbanization, rivers and lakes, and golf courses) were compiled for each watershed also based on publicly available information (Appendix S3). Paddy fields are usually covered by water and dense rice crops and are very different from agricultural lands not used to grow rice, which are relatively dry (i.e., not inundated with water). We used *agriculture* to refer only to agriculture that was not rice. Urban area was the summed area covered by buildings, roads, and railways. A coastal and river artificialization index (CRAI) was calculated based on the percentage of artificial revetments (e.g., concrete‐sealed banks and tetrapods) along coastlines and riverbanks, determined using Google Earth Pro (version 7.3.2.5776 [Map data: Google, Digital Globe]). The CRAI was based on a total of 13 km of coastline and riverbank: 3 km of coastline (i.e., 1.5 km on both sides of the river mouth) and a 10 km of the riverbank (i.e., 5 km upstream on both sides of the river).

Physicochemical data were measured or compiled from several publicly available databases (Appendix S4). The water surface temperature (ST) and salinity were measured in the same sampling bucket immediately after water collection for eDNA analysis, from both high and low tides, with a handheld water‐quality analyzer (LAQUA act ES‐71, HORIBA, Kyoto). Because there was overall no significant difference in ST and salinity between high and low tides apart from three sites with large variations in salinity between tides (Appendix S5), the average ST and salinity between tides were used for later analyses. Data regarding river length, river discharge, surface water dissolved oxygen (DO_s_), bottom water dissolved oxygen (DO_b_), pH, total nitrogen (TN), and suspended solid (SS) were also obtained from publicly available national or regional databases (Appendix S4). We selected data collected from the most downstream observatory station in each river and calculated a 5‐year average from 2014 to 2018 for June–August. We selected the minimum DO_b_ values for the summer season (June–September) from the most recent year for which data were available (from 2016 to 2018). We measured DO_b_ from June to September 2018 and 2019 from the Mukawa, Mabechi, Oyodo, Takatsu, Arakawa, and Yoneshiro Rivers with a conductivity‐temperature‐depth instrument (AAQ1183; JFE‐Advantech, Kobe, Japan) because public measurement stations for these watersheds were either nonexistent or too far from each estuary to be relevant.

### Statistical analyses

The species richness matrix (Table 1) was treated in the same way as a species abundance matrix. A partially constrained redundancy analysis (RDA) with all watershed explanatory variables and latitude as a covariable was applied to produce an ordination graph that portrayed Hellinger's distance among estuaries (i.e., based on relative species richness per threat level instead of relative abundance per species) in a space that would best explain the variation among estuaries (Borcard et al., 2018). We assessed the significance of RDA and its axes with 9999 permutation tests. Variance inflation factors (VIFs) and a forward selection procedure were used to remove variables from the RDA when strong dependencies (i.e., collinearities) between explanatory variables were detected. A selection process can only be performed if the global RDA (i.e., including all explanatory variables) is significant. The procedure, in turn, focuses on all variables in the search for the ones that best explain the species richness per threat level (significant and highest adjusted *R* ^2^). The best variables are included in the model until a candidate variable is deemed nonsignificant or until the adjusted *R* ^2^ of the current model is over the value of the adjusted *R* ^2^ of the global model (Borcard et al., 2018). Red‐list categories were represented as vectors, and their contribution to the dispersion of the estuaries in the reduced RDA space was assessed using the equilibrium circle (Borcard et al., 2018). Proportions of land‐use cover, population, and physicochemical variables were used to constrain the ordination and were represented as vectors and fitted to the RDA. We performed 9999 permutations to assess whether these variables fit the ordination.

**TABLE 1:** Number of fish species (S) per Japanese Red List category per site.*

						**Threatened species (TS)**					
		**S**	**DD**	**LC**	**NT**	LP	**VU**	**EN**	**CR**	**TS**	**RL**
**Sites**					**Red Listed (RL)**						
**1**	**– Mukawa**	21	0	14	3	3	0	1	0	4	7
**2**	**– Mabechi**	37	0	29	3	2	0	3	0	5	8
**3**	**– Naruse**	34	0	31	1	1	0	1	0	2	3
**4**	**– Naka**	43	0	40	0	2	0	1	0	3	3
**5**	**– Fuji**	27	0	22	0	2	1	2	0	5	5
**6**	**– Yahagi**	40	0	32	1	1	2	3	1	7	8
**7**	**– Miyagawa**	37	0	29	2	1	1	4	0	6	8
**8**	**– Yamato**	29	0	26	0	1	1	1	0	3	3
**9**	**– Asahi**	31	0	24	2	1	2	2	0	5	7
**10**	**– Niyodo**	47	0	38	3	2	2	2	0	6	9
**11**	**– Hijikawa**	60	0	53	2	2	1	2	0	5	7
**12**	**– Onogawa**	34	0	31	0	1	0	2	0	3	3
**13**	**– Oyodo**	41	0	38	0	1	0	2	0	3	3
**14**	**– Sendai**	47	0	44	0	1	0	2	0	3	3
**15**	**– Kuma**	36	0	29	2	1	1	3	0	5	7
**16**	**– Chikugo**	20	0	12	1	1	2	3	1	7	8
**17**	**– Takatsu**	38	0	28	4	2	2	2	0	6	10
**18**	**– Hino**	43	0	32	2	2	2	3	2	9	11
**19**	**– Kuzuryu**	42	0	35	4	1	1	0	1	3	7
**20**	**– Arakawa**	28	0	21	3	3	1	0	0	4	7
**21**	**– Akagawa**	46	1	36	4	3	1	1	0	5	9
**22**	**– Yoneshiro**	40	1	29	5	3	1	1	0	5	10
	**Total**	186	1	136	14	6	11	14	4	35	49

* Red list categories: DD, data deficient; LC, least concern; NT, near threatened; LP, local population; VU, vulnerable; EN, endangered; CR, critically endangered.

All data discussed here were tested for normality. Means and standard deviations were used to summarize normally distributed data, and means were used only for non‐normally distributed data. A Student's *t* test was performed when particular tide data were normally distributed and of equal variance; otherwise, a Mann–Whitney test was used.

The average occurrence of red‐listed species was calculated in the same manner as species occurrence in a traditional fish diversity study (Zajonz et al., 2019); that is, occurrence represented a detection event per red‐list category and per site. Finally, a generalized linear model (GLM) based on the Poisson model in which count of red‐listed fish species per estuary was the discrete response variable was used to analyze how red‐listed fish species richness was influenced by land‐use cover, population size, and physicochemical parameters of the environment, which were explanatory variables. No interaction terms were included following common practice in the spatial modeling of community data. An Akaike information criterion (AIC) model‐building framework was used to identify the most parsimonious variables in shaping red‐listed fish species richness in estuaries around Japan (Carlos‐Júnior et al., 2020). All statistical tests and data analyses were performed using R 4.0.4 (R Core Team, 2020); statistical significance level was set at 0.05.

## RESULTS

### Fish diversity

A total of 186 fish species, including 7 invasive species, in 132 genera and 62 families were detected across all 22 sites (Figure 1 & Appendix S1). Site‐level species richness ranged from 20 to 60 (mean [SD] = 37.3 [9.2]). *Mugil cephalus* and *Tridentiger* spp. were the only species detected at all sites. Nineteen species were detected at more than half of the sites, whereas a total of 70 species occurred only once but at different sites.

Overall, 35 and 49 species were classified as threatened and red‐listed species, respectively (Table 1). The number of threatened species per site ranged from 2 to 9 (mean = 4.7), and the number of red‐listed species per site ranged from 3 to 11 (mean = 6.7). On average, red‐listed species were detected at three sites (range 1−21), but only three species occurred at more than 10 sites: *Misgurnus anguillicaudatus* (12), *Anguilla japonica* (16), and *Lateolabrax japonicus* (21).

### Ordination and generalized linear model

**FIGURE 2:**
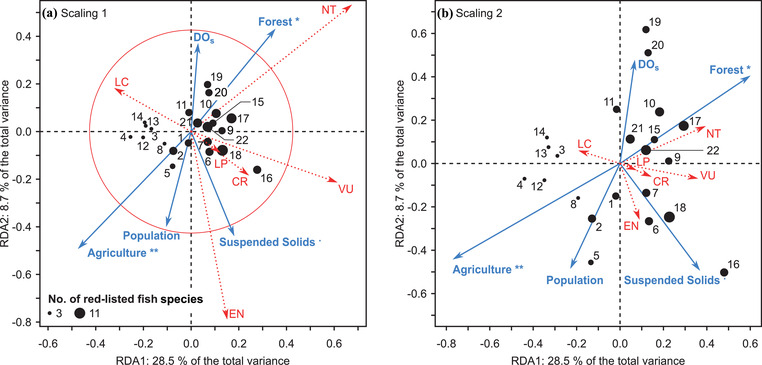
Results of the redundancy analysis (RDA) of the Hellinger‐transformed fish species richness data per Japanese Red List category relative to five explanatory variables for (a) scaling 1 and (b) scaling 2 (point size, proportional to species richness; dotted vectors, red‐list categories; unbroken vectors, explanatory variables; circle, equilibrium contribution; ** *p* < 0.01; * *p* < 0.05; *^.^ p* < 0.1; DO_S_, surface dissolved oxygen; categories: DD, data deficient; LC, least concern; NT, near threatened; LP, local population; VU, vulnerable; EN, endangered; CR, critically endangered). Numbers, river identity [see Figure 1]. A red‐list category vector that is longer than the equilibrium circle radius makes a higher‐than‐average contribution to the ordination in the observed plane

With a moderate cost in explanatory power over a global constrained RDA model (adjusted *R* ^2^ = 71.9%, df = 19, *F* = 7.3, *p* = 0.0243), we produced a partially constrained RDA model (adjusted *R* ^2^ = 43.9% of total variance, df = 5, *F* = 5.3843, *p* = 0.0001) (Figure 2) to represent any major variations in red‐list species richness per site. The partial model remained significant, with no detrimental collinearity (i.e., all VIFs > 2: agriculture = 1.33, forest = 4.32, SS = 1.25, population = 3.9, DO_s_ = 1.57, and latitude = 1.24) relative to the global model, after the forward selection procedure, which removed 14 out of 20 explanatory variables (i.e., variables with a VIF > 10). This partial model was decomposed into two significant canonical axes (*F* = 17.5, *p* = 0.0001 and *F* = 5.36, *p* = 0.0131, respectively), whereas the global model, including all explanatory variables, produced only one significant axis. These first two axes accounted for a large portion (84.9%) of the partially constrained model, representing 37.3% of the total variance.

The triplots (Figures 2a & b) showed that the presence and richness of red‐listed species were significantly correlated with the proportion of forest cover and agricultural cover in watersheds. The red‐listed species richness per site was mainly spread along the first axis (RDA1 28.5% vs. RDA2 8.7% of total variance) following a gradient ranging from small proportions of forest cover and large proportions of agricultural cover to large proportions of forest cover and small proportions of agricultural cover. Sites with a large number of red‐list species, particularly NT and VU species, and to a lesser extent LP and CR species, tended to be associated with high proportions of forest cover and low proportions of agricultural cover. Although not significant, SS seemed to have an effect on the richness of LP, VU, EN, and CR species. Human population and DO_S_ had opposite effects on site ordination but tended to have a rather limited impact on the number of red‐listed species because both vectors were orthogonal to the gradient of the red‐listed species richness (i.e., the first axis).

**FIGURE 3:**
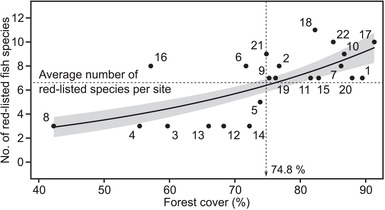
Generalized linear model (GLM) based on the Poisson model with counts of Japanese Red List fish species per estuary as a function of the proportion of watershed area covered by forest (numbers, river identity [see Figure 1]; solid line, regression line; shading, standard error; horizontal dashed line, average number [6.7] of red‐list species; vertical dashed line, threshold of the watershed proportion of forest cover for sites to harbor more red‐listed fish species than average)

Although the overall species richness was not significantly correlated with either forest‐cover or agricultural‐cover proportions in the watersheds (adjusted *R* ^2^ = −0.013, *F* = 0.74, *p* = 0.4 and adjusted *R* ^2^ = −0.046, *F* = 0.079, *p* = 0.78, respectively), the number of red‐listed fish species was positively correlated with the proportion of forest cover in the watersheds (Table 2 & Figure 3). In addition, out of the 15 estuaries harboring more red‐listed species than average (i.e., 6.7 species/site), 13 were located in watersheds with more than 74.8% of forest cover.

**TABLE 2:** Results of the Poisson generalized linear model analysis of the number of fish species on the Japanese Red Lists with the full data set after selection and with only the data set for forest cover (Figure 3)

	Estimate	SE	*z*	*p*
Full data set (AIC 97.368)				
Intercept	–0.517	0.743	–0.697	0.4865
Forest cover	0.030	0.009	3.365	0.0008^a^
Suspended solid	0.017	0.011	1.502	0.1330
Forest cover (AIC 97.446)				
Intercept	0.049	0.606	0.081	0.9358
Forest cover	0.024	0.008	3.130	0.0017^a^

^a^ Significant at 0.01.

## DISCUSSION

A total of 186 fish species and 49 red‐listed species were detected from all 22 sites (Figure 1 & Table 1). These numbers were higher than those from the same 22 rivers (159 fish species and 10 red‐listed species; the sum of species from two recent surveys at the nearest site to each river mouth except the Yamato River, which had only one survey) collected by conventional methods (Ministry of Land, Infrastructure, Transport & Tourism, 2021). Our results support previous reports that indicated that the eDNA metabarcoding method is more effective than conventional sampling methods, particularly for red‐listed species (Deiner et al., 2016; Doi et al., 2021; Nakagawa et al., 2018; Stoeckle et al., 2017). *Mugil cephalus* and *Tridentiger* spp. were detected at all sites. *Mugil cephalus* was widely distributed in the coastal areas of Japan because it is relatively tolerant of polluted waters and a wide range of water temperatures and it is a typical peripheral brackish water fish. *Tridentiger* ssp. was also widely distributed because it shares with *M. cephalus* the characteristics of tolerance of pollution and temperature; it includes two common brackish gobiid fish, *T. obscurus* and *T. brevispinis*; and, when pooled, the genus *Tridentiger* appears to be widespread.

Our results supported the hypothesis that estuarine red‐listed fish richness is significantly influenced by watershed‐scale factors rather than by the estuarine environments. Multivariate ordination showed that the composition of fish species’ threat‐level categories was significantly correlated with the proportion of forest cover and agricultural land area; sites with more red‐listed species were associated with high forest cover and low agricultural land (Figure 2). The GLM showed that forest cover was a good predictor of the number of red‐listed species, but not of the overall fish species richness at the watershed scale (Figure 3). Finally, these results suggest that forest may play a vital role in supporting threatened fish communities. Although the Chikugo River appears to be an exception, with its low forest cover and naturally high suspended solid concentrations (Suzuki et al., 2012), this estuary harbors several continental relict species that are all endangered, mostly endemic, and adapted to this ecosystem (Takita & Yamaguchi, 2009), such as *Boleophthalmus pectinirostris*, *Acanthogobius hasta*, *Salanx ariakensis*, and *Coilia nasus*.

The fact that the proportion of forest cover was overall the only significant variable influencing the number of red‐list species per site indicates that forests play a more vital role in ichthyofaunal conservation, particularly for species at risk, than originally thought. Red‐listed fish species may be more sensitive to watershed‐scale environmental changes than common species. Vasconcelos et al. (2015) analyzed the relationship between fish assemblage composition and environmental characteristics of 430 worldwide estuaries. Their main finding was that species richness differs between marine biogeographic realms and continents. It increases with the mean sea ST, terrestrial net primary productivity, and stability of the connectivity between terrestrial and marine ecosystems. Our results provide novel insights to the results reported by Vasconcelos et al. (2015) and Kume et al. (2021) by showing that land use affects estuarine threatened fish species.

Studies conducted in Canadian streams show that fish species richness and biomass significantly increase as forest cover increases (Stephenson & Morin, 2009). We did not find support for the same trend in our study, likely because the range of forest cover (as a proportion of watershed area) is narrower in Japan (42.3–91.3%) relative to the Canadian study (6–96%) (Stephenson & Morin, 2009). However, despite this narrow range, red‐listed species richness significantly increased with an increasing proportion of forest cover (Figure 3). If the relationship between forest cover and red‐listed fish richness is a general one, then what are the most important mechanisms driving this relationship? Although further mechanistic lines of inquiries are required to address this question, we note some possible factors include forests with abundant water retention capacity mediate the strength of rainfall‐runoff responses and flooding risk that cause large‐scale disruption of the river and coastal ecosystems (Bradshaw et al., 2007; Hayashi et al., 2006); forests prevent land erosion and, therefore, sediment runoff (Onda et al., 2010); and forests are one of the main producers of organically bound iron, which is bioavailable for estuarine and coastal primary production (Onishi et al., 2010). Because iron is one of the major limiting factors for primary productivity in coastal systems, forests are thought to play a certain role in enriching estuarine and coastal habitats with iron (Kawaguchi et al., 1997; Lewitus et al., 2004).

The results of RDA suggest that the composition of fish species at the threat‐level category is significantly correlated with the proportion of agricultural land area. This finding is consistent with that reported by Dauer et al. (2000). They suggest that the proportion of forest cover has the opposite effect of agricultural and urban land‐use cover, which both negatively affect the biodiversity of macrobenthic communities in 10 tributary watersheds of the Chesapeake Bay. In Japan, the main sources of fine sediments that have serious harmful effects on aquatic ecosystems (e.g., Bilotta & Brazier, 2008; Onitsuka et al., 2008; Watanabe et al., 2016) are agriculture, construction work, and domestic and industrial effluents (Siakeu et al., 2004). Agriculture is thought to be the main source of fine sediments and pesticides. The latter, particularly neonicotinoids, which have been heavily used in paddy fields and agricultural lands in Japan, adversely affect fish biodiversity by altering food web structure through the reduction of zooplankton and benthic invertebrate biomass in estuarine areas (Yamamuro et al., 2019), although our results did not indicate an effect of paddy‐field cover on red‐listed species richness.

Although our large‐scale study highlights the value of eDNA metabarcoding for characterizing the presence and absence and richness of red‐listed species, which are often rare or inconspicuous and at risk of extinction without conservation action (IUCN, 2012), there are several caveats in our study and with the use of eDNA metabarcoding. First, we were not able to compare different locations and times of occupancy in one stream with eDNA; hence, eDNA data can be interpreted as an integrated signal (temporally and spatially) of the presence of species in a certain location. Second, although our methods could not detect the influence of land‐use location in the watershed, it is likely that location does matter, and our results should be interpreted with caution. Differences in red‐listed species richness between estuaries are likely related to the location of anthropogenic disturbance along streams. Third, some species listed on the red lists of the JMOE are distributed in particular areas that we did not focus on, such as ponds and upstream part of rivers. Thus, we could not detect all species on the JMOE lists. Finally, further development of this tool is still necessary if one wants to use quantitative information on fish abundance in the natural environment (Brys et al., 2021; Horiuchi et al., 2019; Lafferty et al., 2021). Also, focusing on the types of forest cover, such as unmaintained natural forest, maintained natural forest, and planted forest, could be a next line of investigation that would bring additional valuable information to decision makers.

We found that forests have the potential to support and protect red‐listed fish species in streams and estuaries in Japan. Our results also indicated that the proportion of agricultural cover (and therefore the human population) had a negative effect on red‐listed species. These results may be applicable to other countries that are or will be experiencing deforestation in the coming decades. In addition, because Japan has a relatively high forest cover, these effects may be magnified in countries with less forest cover.

## Supporting information

Supplementary material

Appendix S2: 2015 Census data
Appendix S3: 2014 Land‐use data
Appendix S4: Physicochemical data
Appendix S5: Physicochemical and diversity data comparisons between tides
Appendix S6: Total and average diversity indices, census, physicochemical, and land‐use data
