# Supplementary material for: Effects of forest cover on richness of threatened fish species in Japan
Source: Conserv Biol. 2021 Dec 2;36(3):e13847. doi: 10.1111/cobi.13849 (PMC9299902; doi:10.1111/cobi.13849)
Supplement: Supplementary file 2 — Appendix S2: 2015 Census data Appendix S3: 2014 Land‐use data Appendix S4: Physicochemical data Appendix S5: Physicochemical and diversity data comparisons between tides Appendix S6: Total and average diversity indices, census, physicochemical, and land‐use data [file COBI-36-0-s001.docx]

**Appendix S2: 2015 Census data**

|  | |  |  |  |  |  |  |  |  |  |  |  |  |  |
| --- | --- | --- | --- | --- | --- | --- | --- | --- | --- | --- | --- | --- | --- | --- |
|  | | **Sampling point coordinates** | | | | | | | | |  | **Watershed** | **Population** | **Density** |
| **Watersheds** | | **Latitude** | | | |  | **Longitude** | | | |  | **km^2^** | **Inhabitants** | **Inhabitants/km^2^** |
|  |  |  |  |  |  |  |  |  |  |  |  |  |  |  |
|  |  |  |  |  |  |  |  |  |  |  |  |  |  |  |
| **1** | **– Mukawa** | 42° | 33' | 41'' | N |  | 141° | 55' | 24'' | E |  | 1,249.8 | 9,261 | 7.4 |
| **2** | **– Mabechi** | 40° | 32' | 31'' | N |  | 141° | 30' | 11'' | E |  | 2,036.9 | 176,040 | 86.4 |
| **3** | **– Naruse** | 38° | 22' | 31'' | N |  | 141° | 10' | 22'' | E |  | 1,303.2 | 230,107 | 176.6 |
| **4** | **– Naka** | 36° | 20' | 5'' | N |  | 140° | 35' | 38'' | E |  | 3,275.0 | 906,795 | 276.9 |
| **5** | **– Fuji** | 35° | 6' | 56'' | N |  | 138° | 38' | 1'' | E |  | 3,998.9 | 1,107,088 | 276.8 |
| **6** | **– Yahagi** | 34° | 51' | 28'' | N |  | 136° | 59' | 41'' | E |  | 1,919.4 | 864,934 | 450.6 |
| **7** | **– Miyagawa** | 34° | 32' | 23'' | N |  | 136° | 43' | 22'' | E |  | 924.5 | 128,234 | 138.7 |
| **8** | **– Yamato** | 34° | 36' | 1'' | N |  | 135° | 28' | 16'' | E |  | 1,079.7 | 2,140,267 | 1,982.4 |
| **9** | **– Asahi** | 34° | 36' | 51'' | N |  | 133° | 57' | 46'' | E |  | 1,852.7 | 468,023 | 252.6 |
| **10** | **– Niyodo** | 33° | 27' | 39'' | N |  | 133° | 28' | 53'' | E |  | 1,593.6 | 84,768 | 53.2 |
| **11** | **– Hijikawa** | 33° | 36' | 40'' | N |  | 132° | 28' | 36'' | E |  | 1,229.2 | 90,355 | 73.5 |
| **12** | **– Onogawa** | 33° | 15' | 18'' | N |  | 131° | 42' | 5'' | E |  | 1,501.5 | 217,493 | 144.8 |
| **13** | **– Oyodo** | 31° | 53' | 43'' | N |  | 131° | 27' | 34'' | E |  | 2,283.0 | 574,481 | 251.6 |
| **14** | **– Sendai** | 31° | 50' | 30'' | N |  | 130° | 12' | 32'' | E |  | 1,629.6 | 166,685 | 102.3 |
| **15** | **– Kuma** | 32° | 29' | 2'' | N |  | 130° | 34' | 48'' | E |  | 1,940.5 | 140,425 | 72.4 |
| **16** | **– Chikugo** | 33° | 9' | 39'' | N |  | 130° | 21' | 2'' | E |  | 2,911.5 | 1,058,808 | 363.7 |
| **17** | **– Takatsu** | 34° | 41' | 9'' | N |  | 131° | 49' | 45'' | E |  | 1,112.1 | 31,532 | 28.4 |
| **18** | **– Hino** | 35° | 26' | 54'' | N |  | 133° | 22' | 16'' | E |  | 880.7 | 50,391 | 57.2 |
| **19** | **– Kuzuryu** | 36° | 13' | 5'' | N |  | 136° | 8' | 8'' | E |  | 2,918.0 | 623,348 | 213.6 |
| **20** | **– Arakawa** | 38° | 9' | 7'' | N |  | 139° | 24' | 56'' | E |  | 1,159.7 | 33,972 | 29.3 |
| **21** | **– Akagawa** | 38° | 50' | 52'' | N |  | 139° | 47' | 11'' | E |  | 859.4 | 107,060 | 124.6 |
| **22** | **– Yoneshiro** | 40° | 13' | 30'' | N |  | 140° | 0' | 28'' | E |  | 4,112.2 | 200,956 | 48.9 |
|  |  |  |  |  |  |  |  |  |  |  |  |  |  |  |
|  |  |  |  |  |  |  |  |  |  |  |  |  |  |  |
| **Mean** | |  |  |  |  |  |  |  |  |  |  | 1,898.7 | 427,773.8 | 236.9 |
|  |  |  |  |  |  |  |  |  |  |  |  |  |  |  |

Source: Ministry of Internal Affairs and Communication, Statistics Bureau of Japan. Regional 3^rd^ mesh statistics (2015 Census, population). Only available on DVD (2020).

**Appendix S3: 2014 Land use data**


|  | |  |  |  |  |  |  |  |  |  |  |  |  |  |  |  |  |  |  |  |  |  |  |
| --- | --- | --- | --- | --- | --- | --- | --- | --- | --- | --- | --- | --- | --- | --- | --- | --- | --- | --- | --- | --- | --- | --- | --- |
|  | | **Paddy field** | |  | **Agriculture** | |  | **Forest** | |  | **Abandoned land** | |  | **Urban area** | |  | **River / Lake** | |  | **Golf** | |  | **CRAI** |
| **Watersheds** | | **km^2^** | **%** |  | **km^2^** | **%** |  | **km^2^** | **%** |  | **km^2^** | **%** |  | **km^2^** | **%** |  | **km^2^** | **%** |  | **km^2^** | **%** |  | **%** |
|  |  |  |  |  |  |  |  |  |  |  |  |  |  |  |  |  |  |  |  |  |  |  |  |
|  |  |  |  |  |  |  |  |  |  |  |  |  |  |  |  |  |  |  |  |  |  |  |  |
| **1** | **– Mukawa** | 34.1 | 2.5 |  | 54.1 | 3.9 |  | 1,246.1 | 89.5 |  | 18.3 | 1.3 |  | 14.3 | 1.0 |  | 24.0 | 1.7 |  | 1.4 | 0.1 |  | 16.8 |
| **2** | **– Mabechi** | 105.4 | 4.7 |  | 278.3 | 12.5 |  | 1,713.3 | 76.8 |  | 26.4 | 1.2 |  | 92.4 | 4.1 |  | 15.8 | 0.7 |  | 0.0 | 0.0 |  | 91.5 |
| **3** | **– Naruse** | 329.5 | 23.0 |  | 50.3 | 3.5 |  | 854.5 | 59.7 |  | 17.6 | 1.2 |  | 129.0 | 9.0 |  | 42.3 | 3.0 |  | 7.9 | 0.5 |  | 39.7 |
| **4** | **– Naka** | 579.1 | 16.5 |  | 387.7 | 11.1 |  | 1,943.4 | 55.4 |  | 32.9 | 0.9 |  | 420.6 | 12.0 |  | 88.6 | 2.5 |  | 53.5 | 1.5 |  | 63.2 |
| **5** | **– Fuji** | 162.9 | 3.9 |  | 364.6 | 8.6 |  | 3,118.8 | 73.9 |  | 95.7 | 2.3 |  | 377.4 | 8.9 |  | 80.3 | 1.9 |  | 22.4 | 0.5 |  | 19.8 |
| **6** | **– Yahagi** | 194.4 | 9.2 |  | 63.9 | 3.0 |  | 1,509.5 | 71.7 |  | 17.9 | 0.9 |  | 257.2 | 12.2 |  | 43.8 | 2.1 |  | 18.6 | 0.9 |  | 66.4 |
| **7** | **– Miyagawa** | 47.4 | 4.5 |  | 14.4 | 1.4 |  | 907.1 | 86.2 |  | 8.5 | 0.8 |  | 49.6 | 4.7 |  | 24.1 | 2.3 |  | 0.6 | 0.1 |  | 62.8 |
| **8** | **– Yamato** | 185.4 | 15.4 |  | 46.9 | 3.9 |  | 508.1 | 42.3 |  | 6.8 | 0.6 |  | 412.6 | 34.3 |  | 32.2 | 2.7 |  | 9.7 | 0.8 |  | 96.9 |
| **9** | **– Asahi** | 199.4 | 9.8 |  | 78.3 | 3.9 |  | 1,526.7 | 75.2 |  | 17.4 | 0.9 |  | 154.2 | 7.6 |  | 45.7 | 2.3 |  | 7.4 | 0.4 |  | 82.7 |
| **10** | **– Niyodo** | 61.2 | 3.6 |  | 66.6 | 3.9 |  | 1,488.8 | 86.7 |  | 35.0 | 2.0 |  | 36.5 | 2.1 |  | 26.6 | 1.6 |  | 2.0 | 0.1 |  | 19.9 |
| **11** | **– Hijikawa** | 78.3 | 5.9 |  | 97.2 | 7.3 |  | 1,080.7 | 81.6 |  | 8.2 | 0.6 |  | 41.3 | 3.1 |  | 17.3 | 1.3 |  | 1.6 | 0.1 |  | 65.1 |
| **12** | **– Onogawa** | 161.2 | 10.0 |  | 138.7 | 8.6 |  | 1,101.5 | 68.3 |  | 75.3 | 4.7 |  | 101.1 | 6.3 |  | 30.4 | 1.9 |  | 4.8 | 0.3 |  | 89.6 |
| **13** | **– Oyodo** | 215.8 | 9.0 |  | 277.5 | 11.6 |  | 1,584.4 | 66.0 |  | 35.9 | 1.5 |  | 228.6 | 9.5 |  | 52.6 | 2.2 |  | 6.2 | 0.3 |  | 93.2 |
| **14** | **– Sendai** | 202.9 | 11.6 |  | 107.4 | 6.2 |  | 1,259.5 | 72.2 |  | 16.0 | 0.9 |  | 118.0 | 6.8 |  | 37.0 | 2.1 |  | 2.9 | 0.2 |  | 74.0 |
| **15** | **– Kuma** | 130.5 | 6.3 |  | 62.7 | 3.0 |  | 1,705.9 | 82.8 |  | 49.1 | 2.4 |  | 78.5 | 3.8 |  | 31.2 | 1.5 |  | 2.0 | 0.1 |  | 80.4 |
| **16** | **– Chikugo** | 524.0 | 17.0 |  | 210.3 | 6.8 |  | 1,755.9 | 57.1 |  | 94.6 | 3.1 |  | 390.5 | 12.7 |  | 88.5 | 2.9 |  | 9.5 | 0.3 |  | 94.4 |
| **17** | **– Takatsu** | 43.7 | 3.6 |  | 9.4 | 0.8 |  | 1,111.0 | 91.3 |  | 17.1 | 1.4 |  | 20.0 | 1.6 |  | 15.2 | 1.3 |  | 0.0 | 0.0 |  | 58.2 |
| **18** | **– Hino** | 90.6 | 9.3 |  | 19.4 | 2.0 |  | 802.3 | 82.3 |  | 14.5 | 1.5 |  | 34.1 | 3.5 |  | 12.5 | 1.3 |  | 1.9 | 0.2 |  | 29.4 |
| **19** | **– Kuzuryu** | 371.8 | 12.0 |  | 25.6 | 0.8 |  | 2,356.1 | 76.2 |  | 12.9 | 0.4 |  | 248.3 | 8.0 |  | 72.6 | 2.3 |  | 3.9 | 0.1 |  | 56.8 |
| **20** | **– Arakawa** | 72.8 | 5.8 |  | 9.4 | 0.7 |  | 1,112.9 | 87.9 |  | 19.8 | 1.6 |  | 24.1 | 1.9 |  | 25.8 | 2.0 |  | 0.8 | 0.1 |  | 38.2 |
| **21** | **– Akagawa** | 128.7 | 13.5 |  | 29.6 | 3.1 |  | 712.4 | 74.8 |  | 11.0 | 1.2 |  | 46.6 | 4.9 |  | 22.8 | 2.4 |  | 0.9 | 0.1 |  | 25.8 |
| **22** | **– Yoneshiro** | 303.1 | 7.0 |  | 113.2 | 2.6 |  | 3,698.2 | 85.0 |  | 32.9 | 0.8 |  | 122.0 | 2.8 |  | 77.3 | 1.8 |  | 1.7 | 0.0 |  | 50.6 |
|  |  |  |  |  |  |  |  |  |  |  |  |  |  |  |  |  |  |  |  |  |  |  |  |
|  |  |  |  |  |  |  |  |  |  |  |  |  |  |  |  |  |  |  |  |  |  |  |  |
| **Mean** | | 191.9 | 9.3 |  | 113.9 | 5.0 |  | 1,504.4 | 74.7 |  | 30.2 | 1.5 |  | 154.4 | 7.3 |  | 41.2 | 2.0 |  | 7.2 | 0.3 |  | 59.8 |
| **SD** | |  | 5.3 |  |  |  |  |  | 12.6 |  |  |  |  |  |  |  |  | 0.6 |  |  |  |  | 26.6 |
|  |  |  |  |  |  |  |  |  |  |  |  |  |  |  |  |  |  |  |  |  |  |  |  |

The sum of the land-use areas, including paddy field, agriculture, forest, abandoned land, urban area and golf course for each watershed, was used to calculate the total watershed area on which the watershed proportions of land-use cover (in %) are based. Wetlands were not included in the calculation of the total watershed area as they overlap with other land uses. We preferred using this sum over the total watershed areas data used for population density because differences in the GIS grid resolutions between both data sets might have led to inconsistencies.

CRAI = Coast and River Artificialisation Index

Source: Ministry of Land, Infrastructure, Transport and Tourism. National Land Numerical Information - Land Use Subdivision Mesh (2014)

http://nlftp.mlit.go.jp/ksj/gml/datalist/KsjTmplt-L03-b.html

**Appendix S4: Physico-chemical data**

|  | |  |  |  |  |  |  |  |  |  |  |  |  |  |  |  |  |  |
| --- | --- | --- | --- | --- | --- | --- | --- | --- | --- | --- | --- | --- | --- | --- | --- | --- | --- | --- |
|  | | **Length** | **Discharge *** |  | **Water Surface Temperature** | | |  | **Salinity** | | |  | **Dissolved oxygen** | |  | **pH** | **TN** | **SS** |
|  | |  |  |  | **High tide** | **Low tide** | **Average** |  | **High tide** | **Low tide** | **Average** |  | **Surface** | **Bottom** |  |  |  |  |
| **Rivers** | | **km** | **m^3^/s** |  | **°C** | **°C** | **°C** |  |  |  |  |  | **mg/L** | **mg/L** |  |  | **mg/L** | **mg/L** |
|  |  |  |  |  |  |  |  |  |  |  |  |  |  |  |  |  |  |  |
|  |  |  |  |  |  |  |  |  |  |  |  |  |  |  |  |  |  |  |
| **1** | **– Mukawa** | 135 | 46.9 |  | 20.5 | 19.7 | 20.1 |  | 0.1 | 0.1 | 0.1 |  | 9.3 | 11.2 |  | 7.8 | 0.271 | 8.0 |
| **2** | **– Mabechi** | 142 | 43.1 |  | 23.8 | 23.3 | 23.6 |  | 4.4 | 0.8 | 2.6 |  | 8.8 | 8.2 |  | 7.6 | 1.227 | 14.2 |
| **3** | **– Naruse** | 89 | 30.7 |  | 23.7 | 22.4 | 23.1 |  | 25.5 | 5.4 | 15.5 |  | 8.1 | 6.4 |  | 7.3 | 0.783 | 15.5 |
| **4** | **– Naka** | 150 | 81.2 |  | 25.5 | 27.5 | 26.5 |  | 19.4 | 13.0 | 16.2 |  | 8.5 | 6.0 |  | 7.8 | 1.259 | 5.4 |
| **5** | **– Fuji** | 128 | 53.7 |  | 22.1 | 22.0 | 22.1 |  | 1.5 | 0.7 | 1.1 |  | 9.3 | 7.7 |  | 8.0 | 1.003 | 3.4 |
| **6** | **– Yahagi** | 118 | 32.9 |  | 23.5 | 21.9 | 22.7 |  | 0.1 | 0.1 | 0.1 |  | 8.2 | 4.2 |  | 7.4 | 0.774 | 7.8 |
| **7** | **– Miyagawa** | 91 | 45.7 |  | 23.0 | 22.5 | 22.8 |  | 7.5 | 10.4 | 9.0 |  | 8.1 | 2.5 |  | 7.5 | 0.410 | 9.5 |
| **8** | **– Yamato** | 68 | 23.9 |  | 25.2 | 24.9 | 25.1 |  | 0.1 | 0.2 | 0.2 |  | 9.4 | 0.8 |  | 8.3 | 2.927 | 8.0 |
| **9** | **– Asahi** | 142 | 44.9 |  | 22.7 | 24.2 | 23.5 |  | 4.3 | 6.0 | 5.2 |  | 7.6 | 4.6 |  | 7.8 | 0.566 | 6.5 |
| **10** | **– Niyodo** | 124 | 111.2 |  | 23.3 | 22.7 | 23.0 |  | 4.2 | 0.4 | 2.3 |  | 9.1 | 7.3 |  | 7.6 | 0.385 | 3.5 |
| **11** | **– Hijikawa** | 103 | 38.6 |  | 20.2 | 20.1 | 20.2 |  | 3.3 | 9.1 | 6.2 |  | 7.8 | 7.9 |  | 7.9 | 0.648 | 3.0 |
| **12** | **– Onogawa** | 107 | 98.9 |  | 31.1 | 29.2 | 30.2 |  | 7.8 | 6.6 | 7.2 |  | 7.9 | 7.0 |  | 8.0 | 0.028 | 15.5 |
| **13** | **– Oyodo** | 107 | 144.2 |  | 26.9 | 25.7 | 26.3 |  | 8.2 | 0.7 | 4.5 |  | 8.3 | 4.9 |  | 7.6 | 1.520 | 3.8 |
| **14** | **– Sendai** | 137 | 108.6 |  | 28.7 | 30.0 | 29.4 |  | 22.2 | 6.8 | 14.5 |  | 8.2 | 6.5 |  | 7.5 | 0.840 | 3.6 |
| **15** | **– Kuma** | 115 | 160.2 |  | 29.8 | 28.8 | 29.3 |  | 7.8 | 4.8 | 6.3 |  | 8.4 | 5.9 |  | 7.8 | 0.488 | 4.9 |
| **16** | **– Chikugo** | 143 | 151.7 |  | 31.7 | 33.0 | 32.4 |  | 19.2 | 4.1 | 11.7 |  | 8.0 | 4.4 |  | 7.6 | 1.320 | 43.7 |
| **17** | **– Takatsu** | 81 | 46.6 |  | 26.7 | 24.1 | 25.4 |  | 0.1 | 0.1 | 0.1 |  | 9.5 | 6.4 |  | 7.4 | 0.450 | 4.1 |
| **18** | **– Hino** | 77 | 100.1 |  | 27.4 | 27.3 | 27.4 |  | 0.7 | 0.1 | 0.4 |  | 7.9 | 6.3 |  | 7.3 | 0.648 | 2.7 |
| **19** | **– Kuzuryu** | 116 | 122.0 |  | 26.3 | 24.7 | 25.5 |  | 4.7 | 3.1 | 3.9 |  | 10.8 | 7.3 |  | 8.6 | 0.360 | 7.6 |
| **20** | **– Arakawa** | 73 | 106.5 |  | 24.4 | 22.3 | 23.4 |  | 0.3 | 0.3 | 0.3 |  | 9.0 | 6.9 |  | 7.3 | 0.395 | 8.7 |
| **21** | **– Akagawa** | 70 | 57.3 |  | 24.1 | 26.0 | 25.1 |  | 6.4 | 7.7 | 7.1 |  | 8.8 | 6.0 |  | 7.1 | 1.138 | 7.1 |
| **22** | **– Yoneshiro** | 136 | 89.4 |  | 20.9 | 23.0 | 22.0 |  | 0.1 | 0.1 | 0.1 |  | 8.4 | 6.2 |  | 7.2 | 0.510 | 5.2 |
|  |  |  |  |  |  |  |  |  |  |  |  |  |  |  |  |  |  |  |
|  |  |  |  |  |  |  |  |  |  |  |  |  |  |  |  |  |  |  |
| **Mean** | | 111.4 | 79.0 |  | 25.1 | 24.8 | 25.0 |  | 6.7 | 3.7 | 5.2 |  | 8.6 | 6.1 |  | 7.6 | 0.816 | 8.7 |
| **SD** | | 26.6 |  |  | 3.2 | 3.4 | 3.2 |  |  |  |  |  |  | 2.1 |  | 0.4 |  |  |
|  |  |  |  |  |  |  |  |  |  |  |  |  |  |  |  |  |  |  |

TN = Total Nitrogen and SS = Suspended Solid

Sources:

Ministry of Land, Infrastructure, Transport and Tourism. River Handbook. http://www.mlit.go.jp/river/toukei_chousa/kasen_db/pdf/2019/4-1-4.pdf (2019).

Ministry of Land, Infrastructure, Transport and Tourism. Water Information System. http://www.mlit.go.jp/ (2019).

Miyagi Prefecture Fisheries Research and Development Center. Report of environmental research in Sendai Bay.

Kochi Prefectural Environmental Research Center. Unpublished DO data for the Naruse, Miyagawa, Niyodo, Sendai, Kuzuryu, and Akagawa rivers.

Ministry of the Environment, Japan. Comprehensive Water Environment Information Site. https://water-pub.env.go.jp/water-pub/mizu-site/mizu/kousui/dataMap.asp (2019) for the Naka, Fuji, Yahagi, Yamato, Asahi, Hijikawa, Onogawa, Kuma, Chikugo, and Hino Rivers.

Environmental Science Research Center of Yamagata Prefecture. 2017. Results of Survey of COD and Vertical Distribution of DO in Coastal Sea around Yamagata Prefecture, Ⅱ-type Joint Research by National Institute for Environmental Studies and Local Environmental Research Institute. Japan National Institute for Environmental Studies.

Miyagi Prefecture Fisheries Research and Development Center. 2018. Report of environmental research in Sendai Bay. Available from:

https://www.pref.miyagi.jp/uploaded/attachment/642929.pdf.

Kyushu Electric Power Co. Inc. 2018. Results of sea monitoring. Available from https://www.pref.kagoshima.jp/aj02/documents/64064_20180206201258-1.pdf.

**Appendix S5: Physico-chemical and diversity data comparisons between tides**

|  | **Total** | **High tide** | **Low tide** | ***Statistics*** | ***df*** | ***p* val.** |
| --- | --- | --- | --- | --- | --- | --- |
|  |  |  |  |  |  |  |
| n (sites) | 22 | 22 | 22 | - | - | - |
| **Physico-chemical data** |  |  |  |  |  |  |
|  |  |  |  |  |  |  |
| Average ST (°C) | 25.0 ± 3.2 | 25.1 ± 3.2 | 24.8 ± 3.4 | t = 0.2830 | 41.938 | 0.778 |
| Average salinity | 5.2 | 6.7 | 3.7 | W = 282 | - | 0.351 |
| **Diversity data** |  |  |  |  |  |  |
|  |  |  |  |  |  |  |
| Species richness | 186 | 163 | 143 | - | - | - |
| Average species richness | 37.3 ± 9.2 | 29.7 ± 8 | 28.0 ± 7.2 | t = 0.7298 | 41.550 | 0.470 |
| Threatened species* richness | 35 | 28 | 27 | - | - | - |
| Average threatened species | 4.7 ± 1.7 | 3.8 | 3.6 ± 1.7 | W = 257 | - | 0.727 |
| Red-listed species^†^ richness | 49 | 40 | 39 | - | - | - |
| Average red-listed species | 6.6 | 5.4 ± 2.7 | 5.2 ± 2.4 | t = 0.2382 | 41.503 | 0.813 |
| Average red-listed species occurrence | 3.0 | 3.0 | 3.0 | - | - | - |
|  |  |  |  |  |  |  |

ST = Surface Temperature

* Threatened species are species categorized either as LP, VU, EN or CR.

^†^ Red-listed species are species categorized either as NT, LP, VU, EN or CR.

| **Appendix S6: Total and average diversity indices, census, physico-chemical and land-use data** | |
| --- | --- |
|  |  |
| n (sites) | 22 |
| **Diversity data** |  |
|  |  |
| Species richness | 186 |
| Average species richness | 37.3 ± 9.2 |
| LC species richness | 136 |
| Average LC species | 30.6 ± 9.3 |
| NT species richness | 14 |
| Average NT species | 1.9 ± 1.6 |
| LP species richness | 6 |
| Average LP species | 1.7 |
| VU species richness | 11 |
| Average VU species | 0.95 |
| EN species richness | 14 |
| Average EN species | 1.9 |
| CR species richness | 4 |
| Average CR species | 0.23 |
| Threatened species richness | 35 |
| Average threatened species | 4.7 |
| Red-listed species richness | 49 |
| Average red-listed species | 6.6 |
| Average red-listed species occurrence | 3.0 |
| **Census data** |  |
|  |  |
| Average latitude | 35.7 ± 2.9 |
| Average longitude | 135.8 ± 4.0 |
| Watershed area (km^2^) | 41,771.2 |
| Average watershed area (km^2^) | 1,898.7 |
| Population (inhabitants) | 9,411,023 |
| Average population (inhabitants) | 427,774 |
| Average density (inhabitants/km^2^) | 236.9 |
| **Physico-chemical data** |  |
|  |  |
| River length (km) | 2,452 |
| Average river length (km) | 111 ± 27 |
| Average discharge (m^3^/s) | 79.0 |
| Average ST (°C) | 25.0 ± 3.2 |
| Average salinity | 5.2 |
| Average DO_s_ (mg/L) | 8.6 |
| Average DO_b_ (mg/L) | 6.1 ± 2.1 |
| Average pH | 7.6 ± 0.4 |
| Average TN (mg/L) | 0.816 |
| Average SS (mg/L) | 8.7 |
| **Land use data** |  |
|  |  |
| Average Paddy field (%) | 9.3 ± 5.3 |
| Average Agriculture (%) | 5.0 |
| Average Forest (%) | 74.7 ± 12.6 |
| Average Abandoned land (%) | 1.46 |
| Average Urban area (%) | 7.3 |
| Average River / Lake (%) | 2.0 ± 0.6 |
| Average Golf (%) | 0.30 |
| Average Ramsar (%) | 0.25 |
| Average CRAI (%) | 59.8 ± 26.6 |
|  |  |
| ST = Surface Temperature, DO = Dissolved Oxygen,  TN = Total Nitrogen, SS = Suspended Solid and  CRAI = Coast and River Artificialisation Index | |
